# The Coach’s Eye: A Randomized Repeated-Measure Observational Study Assessing Coaches’ Perception of Velocity Loss During Resistance Training Exercises

**DOI:** 10.1186/s40798-025-00890-1

**Published:** 2025-07-09

**Authors:** Antonio Dello Iacono, Scott Henry, Asaf Ben-Ari, Israel Halperin, Laura Carey

**Affiliations:** 1 Division of Sport, Exercise and Health, School of Health and Life Sciences, Sport and Physical Activity Research Institute (SPARI), University of the West of Scotland, Hamilton, UK; 2 Department of Health Promotion, School of Public Health, Faculty of Medical and Health Sciences, Tel Aviv University, Tel Aviv, Israel; 3 Sylvan Adams Sports Institute, Tel Aviv University, Tel Aviv, Israel

**Keywords:** Autoregulation, Coaching, Gaze strategy, Velocity-based training

## Abstract

**Background:**

Resistance training (RT) coaches regularly instruct their trainees to terminate a set when the repetition velocity drops below a certain threshold, aligned with the principles of velocity loss in velocity-based training (VBT). However, absent of a velocity-tracking device, coaches are required to detect velocity loss through observation—a topic that has never been studied. Here, we assess the accuracy of RT coaches in detecting when trainees reach specific repetition velocity loss thresholds.

**Methods:**

Twenty RT coaches participated in a single experimental session. They observed videos of two trainees completing sets of the barbell bench press and barbell back squat exercises, using three loads (45%, 65%, and 85% of 1 repetition-maximum [1RM]), and recorded from two views (front and side). We asked them to detect when repetition velocity dropped below two velocity loss thresholds (20% and 40% relative to their first repetition). We examined whether load, velocity loss threshold, view, mental fatigue, and gaze strategy (bar or no-bar tracking) influenced accuracy. We compared outcomes using a negative binomial generalized mixed-effects model.

**Results:**

The average absolute error across all conditions was 2.6 repetitions. Coaches improved their accuracy (negative estimates indicate reduced error) when observing a higher velocity loss threshold (40% vs 20%; − 1.8, 95%CI [− 2.3, − 1.3]); observing heavier loads (− 0.8, 95% CI [− 1.5, − 0.1] for 65% 1RM, and − 3, 95%CI [− 3.4, − 2.6] for 85% 1RM compared to 45% 1RM); and employing a bar tracking gaze strategy compared to a no-bar strategy (− 1.7, 95%CI [− 2.7, − 0.4]). In contrast, point of view and mental fatigue had a negligible effect.

**Conclusions:**

While coaches detect velocity loss with some degree of accuracy, their error rates vary depending on the threshold, load, and gaze strategy. These factors should be considered when using perceived velocity loss in practice.

## Key Points

- It is unknown if coaches can accurately detect when their trainees reach repetition velocity loss thresholds.
- Twenty coaches observed videos featuring trainees who performed two exercises with progressively heavier loads (45%, 65%, and 85% of 1RM), recorded from two points of view (front and side).
- Their goal was to detect when repetition velocity decreased more than 20% and 40% relative to the first repetition.
- The average absolute error was 2.6 repetitions across all loads, velocity loss thresholds, views, and strategies.
- Accuracy improved when coaches detected a higher velocity loss threshold, observed exercises with heavier loads, and adopted a bar tracking strategy. In contrast, point of view and mental fatigue had a negligible effect.

## Background

Prescribing resistance training (RT) involves programming various training variables, including exercises, loads, sets, repetitions, and rest intervals [1]. Over the years, numerous fixed and predetermined RT programs have been developed, providing coaches with general recommendations tailored to specific populations and outcomes [2, 3]. For example, a RT program designed to improve muscular strength in novice and intermediate trainees includes 1–3 sets of 8–12 repetitions at 70–85% of one repetition maximum (1RM) [4, 5]. Importantly, fixed and predetermined RT programs typically rely on a single baseline performance measure (e.g., 1RM) taken at the start of a training cycle. While such fixed and predetermined approaches are practical at the group level [3], they lack individualization by failing to account for between- and within-trainee day-to-day performance variability. Several factors, including fatigue [6], sleep deprivation [7], inadequate diet [8], and short-term training adaptations, lead to such variability. Accordingly, fixed and predetermined RT programs do not allow for the frequent adjustments needed to account for consistent performance fluctuations, often resulting in overloading on some days and underloading on others [9].

To address this limitation, RT programs can be personalized through autoregulation frameworks. Autoregulation refers to the adjustment of training variables based on trainees’ actual or perceived performance, aligning with the principle of individualization [10, 11]. A popular autoregulation method is velocity-based training (VBT), in which the loads, number of sets and repetitions are adjusted based on the velocity of repetitions in a set (i.e., lifting speed) [12]. VBT builds upon the strong inverse relationship between load (expressed as kg or %1RM) and movement velocity (m·s^−1^). Moreover, reduced repetition velocity during a set is a clear indication of neuromuscular fatigue [13]. For example, depending on the RT session’s goal, one can adjust the load to achieve a target velocity within a range of repetitions, to ensure velocity does not exceed a specific velocity loss threshold. By measuring velocity in real-time, VBT allows the adjustment of training variables within a session, without the need for reassessing maximal performance (e.g., 1RM testing) or capacity (e.g., maximum repetitions until failure). This makes VBT a more pragmatic approach to individualizing RT programs, and as a result, it has become an appealing method for prescribing, monitoring, and autoregulating RT. However, to implement VBT effectively, valid and reliable velocity-tracking technologies, such as accelerometers, linear position and velocity transducers, and smartphone applications, are required [12]. While these technologies are widely available, portable, and mostly affordable, their use can be impractical in large groups where multiple trainees share the same equipment (e.g., machines or lifting platforms) or when trainees must transition quickly between exercises.

The trainee's perception of velocity has emerged as an alternative to directly measuring repetition velocity [14–19]. In 2014, Bautista and colleagues developed a velocity perception scale to monitor repetition velocity, establishing its concurrent validity against a velocity-tracking device [14]. Subsequent studies have replicated these findings [15, 16], demonstrated acceptable accuracy in perceiving velocity changes relative to the first repetition in a set [18], and found that accuracy improves with feedback on the magnitude and direction of baseline errors [17, 19]. More recently, researchers have explored trainees’ perception of velocity loss as a method to regulate set volume in VBT without velocity-tracking devices [20–22]. This involves trainees subjectively detecting when velocity drops below a fixed threshold (e.g., 20%) relative to the first repetition in a set. For example, Shaw and colleagues asked trainees to verbally report the repetition corresponding to a 20% velocity loss during deadlifts at 60% and 80% of 1RM [21]. Similarly, Dello Iacono and colleagues investigated perceptions of velocity loss at 20% and 40% thresholds across the entire load-velocity spectrum during the barbell bench press [20]. Both studies compared perceived versus actual velocity measurements, finding average absolute errors of about 1.5 repetitions, though with between-individual variability. Finally, da Silva and colleagues found that trainees accurately terminated sets of barbell back squat and bench press exercises within target 15–30% velocity loss ranges in 74% of cases across various loads [22].

While these studies advance the integration of subjective perception of velocity into RT practice, they have only focused on trainees’ perception of velocity loss. In practice, RT coaches also instruct trainees to terminate sets for various reasons, including poor technique, signs of fatigue, and perceived repetition velocity loss, aligning with VBT principles. To provide a more comprehensive understanding of this area, it is essential to also investigate coaches' accuracy in detecting velocity loss.

Therefore, the aim of this study was to assess coaches' accuracy in perceiving repetition velocity loss—hereby referred to as the “coach's eye”. To this aim, we recruited RT coaches and presented them with videos of two trainees performing three sets of two exercises at three incremental loads, recorded from two different views. Coaches were asked to report the repetitions they perceived as corresponding to 20% and 40% velocity loss. Given the lack of evidence on this topic, we employed an observational study design and did not formulate any a priori hypotheses regarding the effects of the variables of interest on the primary outcome. However, we selected the following variables based on their practical relevance and theoretical rationale:

- Velocity loss thresholds of 20% and 40%, as these are the most commonly used in VBT to enhance neuromuscular adaptations.
- Loads corresponding to 45%, 65%, and 85% of 1RM, to represent a broad range of intensities typically prescribed in RT.
- Side and front views, as these are the primary perspectives coaches rely on when making observational judgments during RT.
- We quantified mental fatigue, to explore whether the accuracy of the coach's eye declines throughout repeated tasks.
- We explored coaches' gaze strategies, to examine their association with the accuracy of velocity perception.

## Methods

### Sample Size and Participants

The sample size calculation was based on the precision-for-planning approach described by Cumming [23]. This method requires specifying the target margin of error and the confidence interval of the estimate of interest to compute an accurate sample size. Two key parameters are needed for this calculation: (1) the expected standard deviation (σ) and (2) the magnitude of the parameter estimate (e.g., coach’s eye accuracy) considered practically meaningful in the study's context. Due to the lack of existing data on coaches' accuracy in perceiving repetition velocity loss, we utilized a σ equal to 0.75 repetitions as reported by Dello Iacono and collegaues, who studied perception accuracy among trainees rather than coaches [20]. Based on our judgment and domain expertise in RT, and considering the typical repetition range (4–12) prescribed in such programs, we deemed an accuracy error of 1 repetition as practically meaningful (e.g., corresponding to 8–25% of set volume). The sample size was then estimated using the following formula:

$${\text{N }} = \left( {\frac{{{\mathbf{t}}\_{\text{critical }} \times { }\left( {{\text{N}} - 1} \right)}}{{\text{f}}}} \right)^{2} \times \left( {\chi^{2}_{{{\;\;\text{critical}}}} \times \left( {{\text{N}} - 1} \right)} \right)$$

where: *t*_*_critical*_ is the critical value of the t-distribution at a 95% confidence interval, required because the population (i.e., coaches) standard deviation is unknown. *f* is the ratio between the practically meaningful magnitude (1 repetition) and the standard deviation (σ = 0.75). In this study, *f* was ~ 1.3333. χ^2^_critical_ is the critical value of the chi-square distribution, used to adjust the sample size calculation and increase the assurance (with 99% probability) that the observed margin of error in the new study will not exceed the pre-defined margin.

Based on this calculation, we recruited 20 qualified RT coaches who volunteered to participate in the study (Table 1). Written informed consent was obtained after the participants received an oral explanation of the purpose and potential risks of the study. All procedures were approved by the Ethics Committee of the University of the West of Scotland (approval number: 2024-16384).

**Table 1:** Coaches’ demographics (mean $\pm$ SD)

Age (years)	28.6 $\pm$ 6.8
Sex	18 M and 2F
Hours of RT coaching per week	7.9 $\pm$ 6.6
Experience with VBT (Yes/No)	11/9

*SD* standard deviation, *RT* resistance training, *VBT* velocity-based training

### Procedures

Participants attended the laboratory for a single experimental session. First, we briefed them on the study procedure and obtained informed consent. Next, we assisted the participants in putting on the eye-tracking glasses, calibrated the devices, and familiarized the participants with the study procedures by having them practice the perception of velocity loss tasks with 2 videos—one of the barbell back squat loaded with 65% of 1RM from a side view and the other of the bench press loaded with 45% from the front view—featuring a different trainee than those in the experiment. During the familiarization, they also practiced using the mental fatigue scale. Once familiarization was complete, participants watched 24 videos lasting between 27 and 79 s, in which two trainees (1 male and 1 female) performed two exercises: the barbell bench press and the barbell back squat. Each exercise was performed with three progressively heavier loads: 45%, 65%, and 85% of the trainees’ 1RM, and was recorded from two points of view: front and side. We did not provide feedback on repetition velocity or task accuracy during either the familiarization or experimental tasks.

**Fig. 1:**
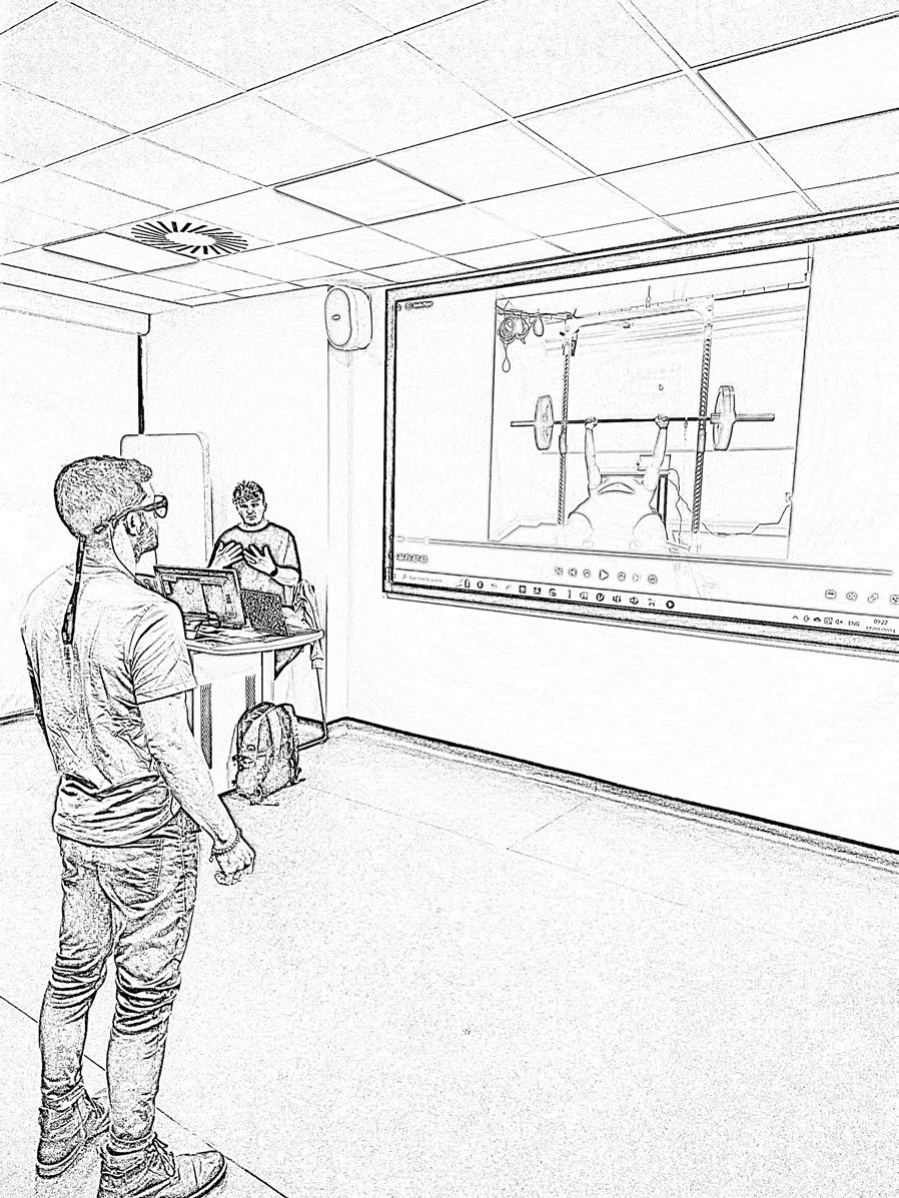
Experimental setup. Participants stood approximately 3 m from the large screen, wearing eye-tracking glasses to track their gaze. The researcher, seated quietly in the corner of the room, controlled the display on the screen, ensuring minimal distraction while managing the experimental setup

The upward (concentric) phase of each repetition was performed as quickly as possible, and the set was terminated at self-assessed task failure—defined as when the trainee could no longer or was unwilling to complete another repetition. The order of the videos was randomized with respect to trainees, exercises, and points of view, while the loads were always presented in a fixed sequence: 45% 1RM first, 65% 1RM second, and 85% 1RM last. The latter information was provided to participants before the videos of each set began. The videos were projected onto a large screen (4 × 1.5 m) in a quiet environment with the audio muted. Participants stood 3 m from the screen and were instructed to indicate (by saying “stop”) the repetition at which they detected a 20% or 40% decrease in bar velocity compared to the first repetition (Fig. 1). Not all videos included both 20% and 40% velocity decreases; some included only a 20% decrease, while others included neither. This information was provided to participants before the session began. Immediately after each answer, or at the end of the video if no answer was given, the researcher paused the video, noted the repetition number, and asked participants to rate their perceived mental fatigue using a visual analog scale. Participants then had a standardized 30-s break before resuming the experiment. The duration of the experiment varied between 43 and 52 min across participants. At the end of the session, participants provided information about their experience with the VBT method and their weekly coaching hours.

### Trainees

The two trainees featured in the videos had at least 5 years of RT experience and routinely trained at least three times per week. They reported to a laboratory for a single session, which included a 1RM assessment for the barbell back squat and barbell bench press exercises, followed by three sets of each exercise loaded at 45%, 65%, and 85% of 1RM and performed to task failure (see ‘Procedures’ section). The 1RM test and subsequent lifting tasks were conducted with a free 20-kg barbell (Origin, Edinburgh, United Kingdom) and calibrated weight plates (Eleiko, Halmstad, Sweden). Trainees did not use any lifting equipment such as shoes, belt, knee pads, or straps. Trainees were instructed to avoid intense RT sessions involving the same exercises within 24 h before testing to prevent muscle soreness or performance decrements. Additionally, they were asked to refrain from heavy meals, caffeine, or ergogenic supplements for at least 2 h before the session.

At the beginning of the session, the trainees completed an 8-min general warm-up consisting of 5 min of individualized, self-selected warm-up on either a cycle ergometer or arm ergometer, followed by 3 min of dynamic stretching for upper and lower body muscles. This was followed by an exercise-specific warm-up, which involved 2–4 sets of 2–3 repetitions each, with progressively heavier loads (velocity range: 1.4–0.3 m·s^−1^). The 1RM for both exercises was then estimated following the protocol described by Loturco and colleagues [24].

After a 10-min rest, the trainees performed three sets of each exercise to task failure, starting with the heaviest load and progressing to the lightest (i.e., 85%−65%−45% 1RM). They were instructed to perform the concentric phase of each repetition as quickly as possible, while maintaining a controlled descent of approximately 2 s until touching a bench (barbell back squat; adjusted to ensure a knee angle of 80–90°) or their chest (barbell bench press). An 8-min rest period was provided between sets and exercises. Barbell velocity during the concentric phase of each repetition was recorded using a linear encoder (Chronojump, Barcelona, Spain) attached with a string tether to the barbell and perpendicular to the ground.

### Video Recordings

Video recordings were captured at an acquisition rate of 25 frames per second, using two full high-definition (Video Resolution: 1920 × 1080 pixels) digital video cameras (Casio Exilim 100, Casio, Japan) mounted on tripods 1.2 m from the ground, and positioned in the frontal (front view) and sagittal (side view at a 90-degree angle) planes, at distances of 3 m and 2 m, respectively. These angles and distances were chosen to: (1) replicate the typical observational positions used by RT coaches in practice, and (2) provide relevant information for analyzing the participants' gaze strategies during the experimental task.

### Mental Fatigue

Participants were asked to report their mental fatigue (“How mentally fatigued are you?”) using a 0 (“no fatigue”) to 100 (“maximal fatigue—I can’t carry on the task”) visual analog scale [25]. An electronic version of the scale was projected on the screen immediately after each answer, and/or at the end of the video in the case the participants provided no answer regarding the perception of velocity loss.

### Eye Tracking

The Tobii Pro Glasses 3 (Tobii Technology, Danderyd, Sweden), lightweight head-mounted glasses (77 g), were used to track coaches' gaze at a sampling rate of 100 Hz. The glasses feature a forward-facing scene camera that records the participant's field of view at 25 frames per second with a resolution of 1920 × 1080 pixels. The glasses were fitted to each participant and calibrated using the Tobii Pro Glasses Controller software (firmware version 1.19.1). Calibration was conducted by instructing participants to focus their gaze on a black dot located on a calibration card, positioned 1 m away.

Post-experiment analysis was performed using Tobii Pro Lab Analyzer software (version 1.24.54542). A built-in fixation filter was applied to the gaze data, which displayed a red circle on the gaze video to indicate fixations. Manual coding was conducted using the gaze video to analyze the concentric phase of each lift. The fixation duration was recorded relative to predefined areas of interest, including the bar, weight plates, lifter’s body, and any other environmental features, such as wall markings. For analysis, these areas were collapsed into two categories: "bar" (the bar, weight plates and ring) and "no bar" (lifter body, environmental feature, any other area). If the participant's fixation was directed at the bar for > 50% of the overall fixation duration for the concentric phase of the lift, it was classified as a "bar strategy." All other remaining trials were classified as “no-bar” strategy.

### Statistical Analysis

The outcome measure in this study—coaches’ eye accuracy—was defined as the absolute error between the detected repetitions (as reported by the coach) and the actual repetitions (measured via the linear encoder) at which velocity exceeded the 20% and 40% loss thresholds. For example, if a 20% velocity loss occurred at the 6th repetition and a coach reported it at the 8th repetition, the absolute error would be 2 repetitions. When a coach did not provide a response regarding detected velocity loss, accuracy was calculated as the absolute difference from the total number of repetitions completed by the trainee. For instance, if a 40% velocity loss occurred at the 12th out of 15 repetitions, and the coach did not indicate any repetition, the absolute error would be 3 repetitions. The same method was applied when neither 20% nor 40% velocity losses were reported. If a coach indicated repetitions exceeding the velocity loss thresholds when such a loss did not occur, the error was calculated similarly. For example, if a coach reported a 40% velocity loss occurring at the 8th repetition when it did not occur at all out of a total of 12 repetitions, the absolute error would be 4 repetitions.

Accuracy was treated as discrete count data, with a lower bound of zero (perfect accuracy) and no theoretical upper bound (indicating lower accuracy). Exploratory data analysis revealed that the data were right-skewed and overdispersed, with variance considerably larger than the mean of the outcome measure. Consequently, we fitted a negative binomial generalized linear mixed-effects regression model, specifying a negative binomial error distribution with a log-link function [26], to examine the odds associated with the predictors: load, view, velocity loss threshold, coaches' gaze strategy, mental fatigue, and order of videos. The following regression model was fitted:

$$\begin{aligned} accuracy_{{in}} = & {\text{ }}b_{{0in}} + {\text{ }}b_{{1 - 3}} load{\text{ }} + {\text{ }}b_{{4 - 5}} view{\text{ }} + {\text{ }}b_{{6 - 7}} velocity{\text{ }}loss{\text{ }}threshold{\text{ }} \\ & + {\text{ }}b_{{8 - 9}} strategy{\text{ }} + {\text{ }}b_{{10}} mental{\text{ }}fatigue{\text{ }} + {\text{ }}b_{{11}} order{\text{ }} + _{{in}} \\ \end{aligned}$$

Here, *accuracy* represented the repeated-measures outcome for each coach, while *load* (a categorical variable with 3 levels: 45% 1RM, 65% 1RM, 85% 1RM), *view* (a categorical variable with 2 levels: front, side), *velocity loss threshold* (a categorical variable with 2 levels: 20%, 40%), and *strategy* (a categorical variable with 2 levels: no-bar, bar) were modelled as fixed effects. *Mental fatigue* and the *order* of videos were included as continuous predictors to account for their effects on the outcome measure. The final regression model also included random intercepts for each coach and random slopes for mental fatigue and strategy to account for the dependencies of each coach, as they provided repeated accuracy ratings. Akaike information criterion score was examined to confirm the selection of the final model to obtain the best-fit model while maintaining model parsimony (see R statistical analysis code script available online at: https://osf.io/sjzrn). Estimated marginal means and 95% confidence intervals (CIs) were calculated alongside comparisons made using post-hoc Tukey adjustments. Significance was set at *P* < 0.05. To validate the assumptions of the negative binomial generalized linear mixed-effects model, tests for uniformity of residuals, outliers and zero-inflation were performed using a simulation-based approach, which confirmed the absence of significant violations of the model fit [27]. All statistical analyses were conducted in R language and environment for statistical computing using the glmer, DHARMa, emmeans, and ggeffects packages, while model assumptions were checked using the performance package (4.0.5; R Core Team, Vienna, Austria). The datasets used in this study are available online at: https://osf.io/m9gds.

## Results

**Fig. 2:**
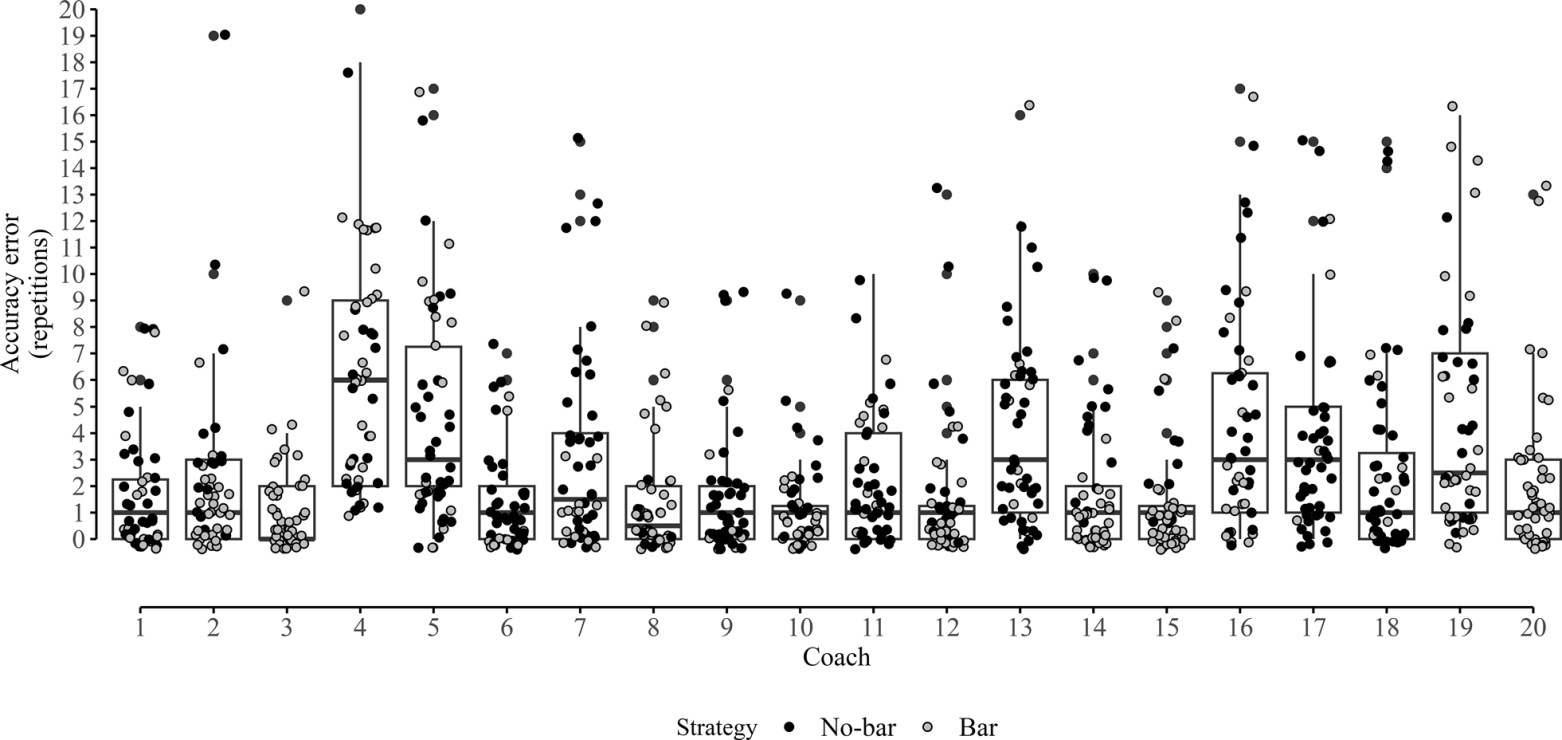
Individual coach accuracy error scores grouped by gaze strategy

Descriptive data from the experimental session are presented as means (SD) and range intervals in Table 2 while the accuracy scores for each coach are illustrated in Fig. 2. The average absolute error was 2.6 repetitions (SD = 3.4; range: 0–20) across all loads, views, velocity loss thresholds, and strategies. To aid interpretation of the results, we present the exponentiated odds and odds ratios of the model outputs as marginal average errors and pairwise comparisons between levels of the fixed effects, expressed in number of repetitions (Table 3). The model’s intercept was estimated at 5.3 (95%CI [3.6, 7.6]), representing the average absolute error in perceiving a 20% velocity loss during sets loaded at 45% 1RM, observed from the front view, using a no-bar tracking strategy. Perceiving repetitions corresponding to a 40% velocity loss, as opposed to 20%, reduced the error by − 1.8 (95%CI [− 2.3, − 1.3]). Videos filmed from the side view had minimal impact on accuracy compared to the front view (− 0.4; 95%CI [− 1.2, 0.5]). Observing heavier loads equal to 65% 1RM and 85% 1RM further decreased the error by − 0.8 (95%CI [− 1.5, − 0.1]) and − 3 (95%CI [− 3.4, − 2.6]), respectively. Using a bar tracking strategy also reduced the error by − 1.7 (95%CI [− 2.7, − 0.4]). Lastly, the model showed trivial effects for both mental fatigue (− 0.1; 95%CI [− 0.1, 0]) and order of video presentation (0.1; 95%CI [− 0.1, 0.1]).

**Table 2:** Descriptive statistics of coaches’ average error across velocity loss thresholds, loads, views and strategies

	Mean (SD); Range
*Velocity loss*	
20%	2.96 (3.18); 0–15
40%	2.26 (3.57); 0–20
*Load*	
45% 1RM	3.69 (4.7); 0–20
65% 1RM	2.79 (2.72); 0–15
85% 1RM	1.35 (1.53); 0–9
*View*	
Front	2.58 (3.39); 0–20
Side	2.64 (3.4); 0–19
*Strategy*	
No-bar	3.06 (3.5); 0–20
Bar	2.15 (3.22); 0–17

*SD* standard deviation

**Table 3:** Negative binomial generalized mixed-effects linear regression model outputs on coaches’ accuracy

Variable	Estimate	95% CI Lower	95% CI Upper	*P* value
Intercept	5.3	3.6	7.6	< 0.001*
Velocity Loss (40% vs 20%)	− 1.8	− 2.3	− 1.3	< 0.001*
View (Side vs front)	− 0.4	− 1.2	0.5	0.389
Load (65% 1RM vs 45% 1RM)	− 0.8	− 1.5	− 0.1	0.034*
Load (85% 1RM vs 45% 1RM)	− 3	− 3.4	− 2.6	< 0.001*
Strategy (Bar vs no-bar)	− 1.7	− 2.7	− 0.4	0.012*
Mental fatigue	− 0.1	− 0.1	0	0.097
Order	0	− 0.1	0.1	0.964
*Random effects*				
σ^2^ = 2.2				
τ _coach_ = 1.5				
τ _fatigue_ = 0				
τ _bar strategy_ = 1.4				
ρ = − 0.41				
N = 20				
Observations = 960				
Marginal R^2^ = 0.204				
Conditional R^2^ = 0.375				

^*^P-value < 0.05

CI: confidence interval

Intercept: 20% VL at 45% 1RM observed from front view using a no-bar tracking strategy

σ^2^: within-subject variability

τ _coach_: between-coach intercept variability

τ _fatigue_: between-coach fatigue variability

τ _bar strategy_: between-coach bar variability

ρ: intercept-slope correlation

## Discussion

We assessed coaches' accuracy in perceiving repetition velocity loss as they observed trainees performing three sets of two exercises at three different loads. The average error was 2.6 repetitions across all velocity loss thresholds, loads, views, and gaze strategies. Accuracy improved when coaches detected a higher velocity loss threshold, observed exercises with heavier loads, and adopted a bar tracking strategy. These findings were consistent across both points of view, with mental fatigue and the order of repeated tasks having negligible impact.

Coaches' accuracy improved when detecting repetitions corresponding to a 40% velocity loss compared to 20% velocity loss. The main reason resides in the repetition-velocity loss profile, which typically develops in a progressive manner during RT exercises. As a set progresses, velocity decreases are greater between consecutive repetitions around higher velocity loss thresholds (e.g., $\ge$ 40%) compared to lower ones (e.g., $\le$ 20%) [28]. Coaches were likely able to perceive this more pronounced decrease in movement velocity, leading to improved accuracy. This reason may also explain the improvement in accuracy under heavier loads, where the velocity loss per additional repetition is even more substantial due to the lower number of repetitions that can be performed in a set [5]. Interpreting the marginal average errors helps contextualize the practical implications of these findings. When observing trainees performing exercises loaded with 45% 1RM, coaches may misjudge a 20% and 40% velocity loss by approximately 5 and 3 repetitions, respectively. These errors are reduced with heavier loads, where errors of 4 and 2 repetitions are expected for 65% 1RM, and 2 and less than 1 repetition for 85% 1RM, respectively. This suggests that a case-by-case judgment would be required when considering the use of perceived velocity loss as an alternative to velocity-tracking technology for implementing the VBT method. For instance, while an error of 1 repetition is likely negligible in sets loaded with 85% 1RM and targeting a 40% velocity loss—if a total of 8 repetitions is expected—an error of 4 repetitions could be meaningful in sets loaded with 65% 1RM and targeting a 20% velocity loss, where only 6 repetitions are prescribed. Therefore, coaches must determine whether their error rates are acceptable based on their overall RT goals.

We found that using a bar tracking strategy significantly improved accuracy rates, reducing the absolute error by approximately 2 repetitions. We speculate that coaches may have better detected key information regarding bar or weight plates’ position, displacement, and velocity, leading to enhanced accuracy. Notably, coaches were not given any instructions about which strategy to use before the study, nor did they receive feedback during the experimental tasks. Most coaches alternated between no-bar and bar tracking strategies randomly, without a systematic pattern across exercises, loads, velocity loss thresholds, or views (Fig. 2). It is likely that accuracy would have improved even further if the coaches had received clear instructions to consistently use the bar tracking strategy and had practiced it during the familiarization videos. The analysis of random effects (Table 3), examining the within-coach relationship between the intercept (i.e., no-bar tracking strategy estimate) and the slope (i.e., bar vs. no-bar tracking strategy difference), supports this assumption [29]. The negative correlation coefficient (ρ =  − 0.41) between intercept and slope suggests that coaches who were less accurate with the no-bar tracking strategy (i.e., higher intercept) showed greater improvement when switching to a bar tracking strategy (i.e., larger negative slope). Collectively, these findings are encouraging, as they indicate that with clear instructions and training, an optimal strategy for perceiving velocity loss can be learned and refined.

To the best of our knowledge, we are the first to assess coaches' accuracy in detecting trainees' velocity loss. Although no previous studies have directly investigated this, our findings can be compared to research examining similar tasks, such as how accurately trainees perceive their own velocity loss or how coaches estimate the number of repetitions left before task failure (i.e., repetitions in reserve). For instance, Dello Iacono and colleagues assessed trainees' accuracy in detecting 20% and 40% velocity loss during the bench press, reporting an average absolute error of 1 repetition [20]. Similarly, Shaw and colleagues examined 20% velocity loss detection during deadlifts at 60% and 80% 1RM, with an average error of 1.4 repetitions [21]. In contrast, we observed a larger average error of 2.6 repetitions, suggesting trainees may be more accurate than coaches. However, our findings showed coaches’ accuracy improved with heavier loads, whereas Dello Iacono and colleagues reported a decline under the same conditions [20], and Shaw and colleagues observed no difference [21]. We also found higher accuracy at the 40% threshold, whereas Dello Iacono and colleagues found no effect of threshold [20]. This strengthens our recommendation for a case-by-case judgment when considering the use of perceived velocity loss as an alternative to velocity-tracking technology. Another relevant comparison is with Emanuel and colleagues, who examined coaches' accuracy in estimating repetitions from failure [30]. As with our study, they found that coaches' accuracy improved with heavier loads. Emanuel and colleagues also explored the role of coaching experience and found that it had no significant impact on accuracy. Unfortunately, we were unable to evaluate the effect of experience with VBT due to our small sample size and discuss this further along with other limitations of our study.

This study has several limitations worthy of discussion. First, coaches usually observe trainees performing exercises live rather than on a screen. Future research should explore how well coaches can detect repetition velocity loss in RT settings rather than relying on video recordings. Second, the calculation method for absolute error when the velocity threshold was not exceeded may underestimate the true error. If a coach incorrectly judged the threshold to be reached near the end of a set, the resulting error might appear small due to the few remaining repetitions, even though the trainee could have performed more. This calculation approach does not account for how many additional repetitions could have been completed before exceeding the velocity threshold. However, since trainees terminated the set voluntarily the true extent of the error remains unknown. Third, we were unable to examine the effects of VBT experience on accuracy rates due to the small sample size. However, it is important to note that coaches with experience in VBT may not necessarily have an advantage over those without such experience. Typical VBT practice relies heavily on tracking devices that provide immediate augmented feedback. This can limit opportunities for coaches to refine their observational skills, potentially diminishing the benefits of VBT experience in accurately perceiving velocity loss during RT. The findings of Emanuel and colleagues regarding the trivial effect of experience on accuracy further support this notion [30]. Finally, the videos included only two exercises, with coaches asked to detect velocity loss at 20% and 40%. Therefore, it remains unclear whether our findings can be generalized to other exercises and velocity loss thresholds.

## Conclusions

This study is the first to investigate coaches' accuracy in perceiving repetition velocity loss. We found that average accuracy errors ranged from 1 to 5 repetitions across all conditions. Accuracy improved when coaches detected a higher velocity loss threshold, observed exercises with heavier loads, and adopted a bar tracking strategy. These findings suggest that coaches can utilize perception of velocity loss to implement the VBT method, provided they carefully consider the expected error rates in relation to their overall RT goals.

## Data Availability

The datasets used in the current study, and the R statistical analysis code script, are available online at: https://osf.io/ha8ke/files/osfstorage.

## Abbreviations

RT: Resistance training
VBT: Velocity-based training
1RM: 1 Repetition-maximum
CI: Confidence interval
SD: Standard deviation
